# Achieving Ultra-Reliable Low Latency Communication in 5G and Beyond

**DOI:** 10.3390/s26113485

**Published:** 2026-06-01

**Authors:** Rojeena Bajracharya, Rakesh Shrestha

**Affiliations:** 1 Department of Computer Science and Engineering, Mälardalen University, 72123 Västerås, Sweden; 2 Research Institute of Sweden (RISE), 72215 Västerås, Sweden; rakesh.shrestha@ri.se

**Keywords:** URLLC, 5G, 6G, latency, reliability, coexistence

## Abstract

Ultra-reliable low-latency communication (URLLC) is a fundamental technology that plays a crucial role in enabling fifth-generation new radio (5G-NR) communication. URLLC aims to provide a highly reliable connection with strict block error probability requirements and extremely low latency for mission-critical and remote operations. Meanwhile, the advent of sixth-generation (6G) communication, marked by its novel, immersive, and high-stakes control applications, imposes notably more stringent demands on reliability and latency, alongside the added requirements of high data rates, scalability, precision, security, and real-time operation. This scenario introduces unparalleled challenges for both system architecture and the solutions it entails. Several previously proposed solutions, such as retransmission schemes, error correction techniques, and grant-free access, have been insufficient for emerging requirements, as most of these solutions primarily facilitate either low latency or high reliability, but not both. Latency and reliability are conflicting objectives of URLLC. Therefore, an in-depth understanding of the associated issues and careful mitigation of these challenges are essential. This article provides an extensive review of 5G URLLC, emphasizing its technical evolution from 3GPP Release 15 through 19, while also detailing its inherent shortcomings and the potential solutions required for 6G and beyond. We investigate the prerequisites and enabling technologies necessary for URLLC services, exploring related issues across various network components, including frame structure, propagation, processing, retransmission, scheduling, fading, and interference. An important discussion is provided on the fundamental trade-off between latency and reliability, particularly due to retransmission mechanisms. Furthermore, we examine the practical limitations of 5G URLLC when coexisting with other 5G application use cases, such as enhanced mobile broadband (eMBB) and massive machine-type communication (mMTC). Finally, we discuss the future trajectory of URLLC in 6G, identifying key research challenges and opportunities to meet the escalating demands of future mission-critical applications.

## 1. Introduction

The fifth-generation (5G) wireless communication system has transformed mobile networking beyond traditional improvements in data rates and coverage by introducing a flexible service-oriented architecture capable of supporting heterogeneous applications and connectivity requirements. To address diverse communication scenarios, 5G defines three major service categories: enhanced mobile broadband (eMBB), massive machine-type communication (mMTC), and ultra-reliable low-latency communication (URLLC). Among these, URLLC represents one of the most transformative features of 5G, enabling mission-critical applications that require extremely low end-to-end (E2E) latency and ultra-high reliability [1,2]. Specifically, URLLC targets latency below 1 ms and reliability levels exceeding 99.999%, making it essential for applications where communication failure or delay may lead to severe operational or safety consequences.

URLLC is expected to revolutionize several sectors, including industrial automation, autonomous transportation, healthcare, public safety, and smart infrastructure. In Industry 4.0 and Industry 5.0 environments, URLLC enables wireless factory automation, intelligent robotics, and real-time machine coordination with stringent latency and reliability constraints [3,4]. Similarly, autonomous driving and Vehicle-to-Everything (V2X) communication require highly reliable and low-latency connectivity to support collision avoidance, cooperative driving, and intelligent transportation systems [5,6]. In healthcare, URLLC facilitates remote surgery, teleoperation, and tactile Internet applications through real-time robotic control and haptic feedback [7,8]. Beyond mission-critical services, URLLC also supports immersive applications such as cloud gaming, augmented reality (AR), extended reality (XR), smart grids, defect detection, and live event broadcasting.

Despite its significant potential, achieving URLLC requirements in practical wireless systems remains highly challenging. The stringent latency and reliability targets cannot be satisfied solely through isolated PHY-layer optimization but instead require end-to-end system-level enhancements across radio access, fronthaul/backhaul networks, edge computing, and core infrastructure [9,10]. Consequently, extensive research and standardization efforts have been conducted to improve URLLC performance through advanced scheduling [11], short transmission time interval (TTI) design [12], grant-free access [13], beamforming and spatial diversity [14], network coding [15], caching [16], multi-connectivity [17], non-orthogonal multiple access (NOMA) [18], machine learning-based optimization [19], and network slicing [20]. However, many existing solutions primarily optimize either latency or reliability independently, while simultaneously satisfying both objectives remains difficult due to their conflicting nature [21,22].

**Figure 1:**
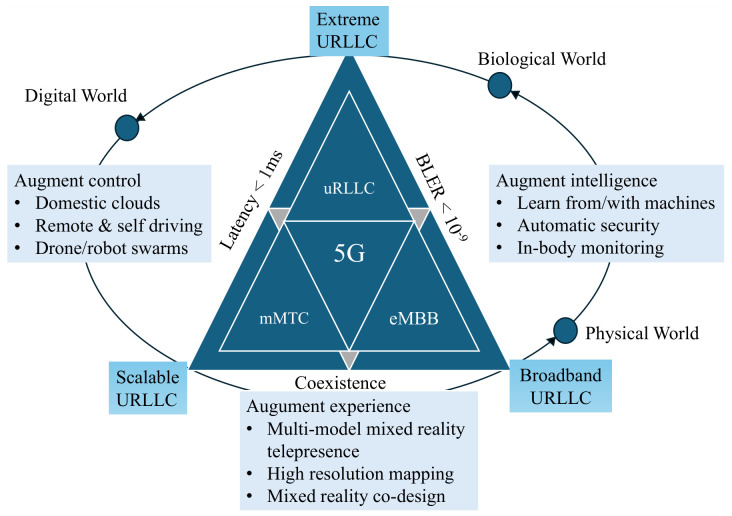
Representative 6G URLLC service categories and application scenarios.

The evolution toward sixth-generation (6G) communication systems further intensifies URLLC requirements beyond current 5G capabilities, as shown in Table 1. According to the ITU IMT-2030 vision [23], 6G is expected to provide intelligent, ubiquitous, and AI-native communication systems integrating sensing, computing, sustainability, and immersive digital experiences. In this context, URLLC evolves toward hyper-reliable low-latency communication (HRLLC), targeting sub-millisecond latency and reliability beyond $10^{-9}$. Figure 1 illustrates the transition from the conventional 5G service pillars (eMBB, mMTC, and URLLC) toward emerging 6G communication domains. Future applications such as Industry 5.0 automation, autonomous mobility, immersive XR, digital twins, telepresence, remote robotic surgery, and resilient critical infrastructure require deterministic communication with ultra-high precision and reliability [24,25,26]. To support these demanding services, 6G introduces extreme URLLC, scalable URLLC, and broadband URLLC, enabled by advanced technologies including AI-driven orchestration, integrated sensing and communication (ISAC) [27], reconfigurable intelligent surfaces (RISs) [28], terahertz (THz) communication [29], and multi-access edge computing (MEC) [30,31,32].

However, existing 5G URLLC capabilities remain insufficient to fully support future 6G requirements involving massive connectivity, immersive applications, and deterministic real-time communication. In addition, coexistence among URLLC, eMBB, and mMTC services introduces complex trade-offs in resource allocation, scheduling, interference management, and scalability. These challenges necessitate a comprehensive system-level understanding of URLLC evolution, standardization progress, deployment constraints, and cross-layer optimization strategies for future 6G systems.

The remainder of this paper is organized as follows. Section 2 presents a literature review. Section 3 introduces URLLC and its service requirements, emphasizing the stringent latency and reliability demands needed to support immediate operational applications. Section 4 summarizes the essential technologies and ongoing standardization efforts related to 5G, Beyond 5G (B5G), and 6G URLLC. Section 5 examines the main sources of end-to-end delay and reliability degradation in wireless networks and reviews previously proposed mitigation techniques. This section also discusses the fundamental trade-off between latency and reliability in URLLC systems. Section 6 presents the vision of URLLC Beyond 5G. Section 7 addresses the coexistence challenges and potential solutions associated with integrating URLLC with other 5G services, including eMBB and mMTC. Finally, Section 8 outlines possible evolutionary pathways for URLLC in 6G networks and highlights open research directions.

## 2. Related Work

Existing studies on URLLC have extensively investigated techniques for achieving stringent latency and reliability requirements in 5G and beyond networks. Early research primarily focused on PHY- and MAC-layer enhancements [1,2,34,35,36]. Various methods have been introduced to jointly improve latency and reliability through efficient scheduling [11], short TTI design [12], beamforming and spatial diversity [14], network coding [15], caching [16], multi-connectivity [17], grant-free access [13], and non-orthogonal multiple access (NOMA) [18]. Furthermore, machine learning and network slicing have been explored for intelligent resource management and service customization [19,20].

Several survey papers have summarized URLLC challenges and enabling technologies for 5G and emerging 6G systems. For example, ref. [25] provides a layered overview of URLLC evolution and enabling technologies, whereas [37,38] focus on AI/ML-driven optimization and security aspects, respectively. Other works mainly emphasize PHY/MAC-layer techniques [1,34,35], specific vertical applications such as healthcare and industrial automation [39], or individual performance metrics including latency–reliability trade-offs [25].

Despite these contributions, several research gaps remain in the current literature. Most existing works analyze latency and reliability independently without comprehensively investigating their interdependence across the end-to-end network stack [2,26]. Moreover, coexistence among URLLC, eMBB, and mMTC services is generally discussed qualitatively, with limited system-level analysis of resource sharing, scheduling conflicts, and performance trade-offs. Practical deployment challenges, including fronthaul/backhaul limitations, mobility management, synchronization constraints, and large-scale network integration, are also insufficiently addressed.

With the transition toward 6G, recent research has highlighted new requirements such as sub-millisecond latency, immersive XR, autonomous mobility, integrated sensing, AI-native communication, and ultra-high reliability [23,24,25,40,41]. Emerging technologies including AI/ML-driven solutions, MEC and digital twins are increasingly recognized as key enablers for future URLLC systems [28,31,32,42]. However, a comprehensive survey jointly covering standardization evolution, coexistence analysis, cross-layer design, practical deployment issues, and future 6G URLLC research directions remains limited in the current literature [41,43]. Table 2 summarizes the comparison between existing URLLC surveys and the proposed work in terms of coverage, technical depth, coexistence analysis, standardization discussion, and practical deployment considerations. Motivated by these gaps, this paper provides a comprehensive and system-level survey of URLLC evolution from 5G to 6G.

## 3. Definitions and KPI

URLLC is defined using two indicators: latency and reliability. Latency is evaluated based on the round-trip time (RTT) or E2E latency, and reliability is evaluated by measuring the successful transmission rate of data packets of a specific size within a specified period of time (i.e., measuring BLER) [2].

### 3.1. Latency

Latency refers to the delay a packet experiences from the moment it enters a protocol layer at the transmitter until it exits the same layer at the receiver [44]. E2E latency encompasses various delays, including propagation delays, queuing delays, processing and computing delays, and any retransmission delays [45]. For URLLC, the minimum user plane latency requirement is less than 1 millisecond.

The E2E latency can be decomposed into multiple components as follows:

(1) $$T_{E2E}=T_{tx}+T_{prop}+T_{proc}+T_{reTx}+T_{queue}$$

where $T_{tx}$ is the transmission time interval; $T_{prop}$ corresponds to the propagation delay over the wireless channel; $T_{proc}$ denotes the processing delay, including encoding, decoding, and scheduling operations; $T_{retx}$ is the time required for retransmission. $T_{queue}$ represents the queuing delay at the transmitter or intermediate nodes;

For short-packet transmission, the transmission delay can be expressed as:

(2) $$T_{tx}=\frac{L}{R}$$

where *L* is the packet size (in bits) and *R* is the achievable data rate (in bits per second). This decomposition highlights how latency reduction techniques operate across different layers of the system. Mini-slot transmission reduces $T_{tx}$ by shortening the transmission interval, allowing packets to be sent more frequently without waiting for a full slot. Grant-free access reduces $T_{queue}$ by eliminating the need for scheduling requests and grants, enabling immediate transmission for time-critical packets. Optimized signal processing algorithms, such as fast encoding and decoding, reduce $T_{proc}$ by accelerating data preparation and recovery at both transmitter and receiver. Multi-connectivity or packet duplication reduces $T_{prop}$ by providing multiple transmission paths simultaneously, lowering the probability of delay caused by poor channel conditions.

### 3.2. Reliability

Reliability is defined as the probability that a specific volume of data, denoted as *D*, will be successfully transmitted within a time frame of *T* [44]. A key reliability criterion is that the probability of successfully delivering a 32-byte layer two protocol data unit within 1 millisecond must be at least $1\times 10^{-5}$. Reliability $P_{rel}$ in URLLC is typically defined in terms of outage probability:

(3) $$P_{rel}\left(\theta \right)=1-P_{outage}\left(\theta \right)$$

where $P_{\mathrm{outage}}\left(\theta \right)$ is defined as the probability that the instantaneous channel capacity falls below the required transmission rate, and depends on system parameters such as SNR, blocklength, and fading characteristics. The overall reliability is influenced by multiple factors, including transmission errors caused by channel fading, packet loss due to collisions or interference, queuing delays that exceed the allowed latency, proactive packet dropping, and network configuration parameters such as scheduling policies, resource allocation, and multi-connectivity strategies. Assuming these factors are independent, the total reliability can be approximated as the product of the probabilities of successfully avoiding each type of failure.

### 3.3. Performance Metrics and KPIs

Unlike earlier generations such as 2G, 3G, and 4G, where capacity and data rates were primary concerns, URLLC shifts the focus to reliability and latency. Moreover, most previous studies on reducing average delay through the performance evaluation of throughput–delay trade-offs [46], delay-limited link capacity [47], or network effective capacity [48] are not valid for URLLC. URLLC demands average worst-case latency rather than average delay. Similarly, in the network layer, an abundance of research on queue-based resource allocation and optimization of its effective capacity is available [49,50,51,52]. However, stability is a crucial concern for queuing networks; the delay distribution and probabilistic bounds in queue networks are hardly addressed.

Table 3 offers a summary of the worst-case latency and reliability specifications for different usage scenarios of URLLC in mission-critical applications. The latency and reliability values reported are aggregated from literature sources and represent application-dependent design targets rather than standardized requirements. Some values, particularly for extreme reliability levels, reflect proposed or aspirational targets for future mission-critical communication systems. For example, remote surgery requirements vary significantly depending on the level of teleoperation (e.g., haptic control vs. supervisory assistance), with reported latency targets ranging from sub-10 ms to approximately 100 ms in the literature [53,54]. Table 3 shows that the majority of URLLC verticals, particularly those in manufacturing, place a strong priority on reliability, whereas for real-time applications, the priority shifts to low latency. Industrial robots and mobile robots pose the toughest demands for wireless communications, as sub-millisecond latency and ultra-high reliability are essential to avert risks to manufacturing safety and productivity. Automated guided vehicles and handheld terminals similarly require rapid and dependable interactions to enable precise remote operation and real-time material logistics within dynamic industrial environments. In contrast, sensors and security cameras can withstand higher latency and benefit from slightly relaxed reliability, as their primary role centers on periodic data reporting or non-critical surveillance. Augmented reality (AR)/virtual reality (VR) devices and head-mounted displays, used in industrial and healthcare settings, leverage low-latency for seamless and immersive experiences, where delays could disrupt operational flow. Critical applications such as V2X vehicular communication and remote surgery elevate both latency and reliability requirements even further, ensuring immediate, fail-safe coordination and intervention in life-sustaining scenarios.

## 4. Evolution and Standardization of URLLC

URLLC has evolved from early concepts of ultra-reliable communication (URC) and parallel interface communications introduced in the mid-1990s, initially developed for high-speed point-to-point links over copper and optical fiber with extremely low error rates [25]. Early work on ultra-reliability focused on optical networks using diversity techniques to achieve minimal error rates, while latency demands were initially less stringent and treated independently [59,60]. The wireless communication community began formal discussions on URC around 2011, addressing reliability through diversity schemes but with comparatively relaxed latency requirements termed “real-time communication” [25,61]. Over time, applications demanding both ultra-high reliability and low latency emerged, notably industrial automation, remote surgery, and autonomous vehicles, driving the joint consideration of these factors under the 5G URLLC umbrella.

**Figure 2:**
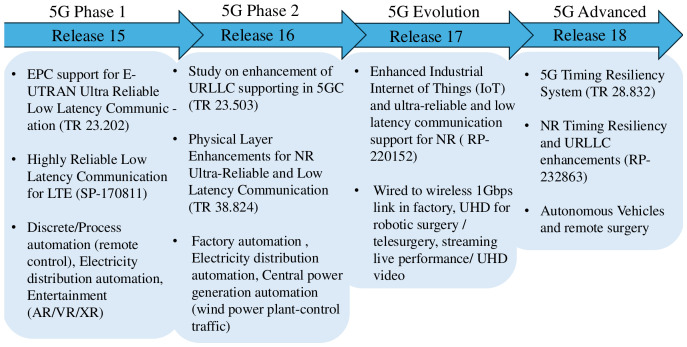
Standardization phases for 5G URLLC in 3GPP.

The advancement of URLLC within the 3GPP framework has unfolded across various releases, each introducing novel features, optimizations, and enhancements tailored to the exacting demands of URLLC applications. Before Release 14 (at the beginning of 2014), the considerations of reliability and latency were largely treated as distinct entities. However, with the advent of Release 14, a paradigm shift has emerged toward addressing these requirements in tandem. For instance, the emergence of V2X services in the early stages necessitated Long Term Evolution (LTE) radios to accommodate stringent latency demands, such as “Message transfer latency no longer than 100 ms with 20 ms maximum allowed latency in some specific use cases” [62], all while maintaining uncompromising reliability. Initiatives like the “Enhancements of NB-IoT” [63] and efforts within legacy 2G technologies like the Global System for Mobile Communications (GSM)/Enhanced Data rates for GSM Evolution (EDGE) radio access network, were already underway to mitigate latency. In parallel, features to boost reliability, such as “Extended architecture support for cellular Internet of Things,” sought to do so without raising latency [64]. Figure 2 and Table 4 provide a brief overview of the key 3GPP releases relevant to URLLC, which are described below.

### 4.1. 3GPP Release 15

- Release 15 marks the initial 5G phase in which URLLC-capable mechanisms were introduced mainly through LTE-based and early 5G system support, laying the foundation for later URLLC standardization [65].
- The main technical contributions include the following:
  1. Reduced transmission and processing delay through short TTI support, early data transmission, uplink data compression, and faster hybrid automatic repeat request (HARQ) timing. These changes reduced the number of scheduling and retransmission steps needed before data delivery.
  2. Grant-free transmission support, which allowed selected uplink packets to be transmitted without waiting for explicit grants, thereby lowering access delay for small and time-sensitive payloads.
  3. Reliability-oriented enhancements such as PDCCH (physical downlink control channel) repetition, semi-persistent scheduling repetition, packet duplication at the packet data convergence protocol (PDCP) layer, and tighter control-format configuration. These mechanisms increased delivery robustness under poor radio conditions.
  4. Improved system access and recovery procedures, including granular time reference support, enhanced master information block and system information block demodulation, and reduced re-synchronization overhead [66]. These features shortened re-acquisition time after disruption.
  5. Evolved Packet Core (EPC) support for E-UTRAN (Evolved Universal Terrestrial Radio Access Network) URLLC, enabling URLLC-style operation in LTE/EPC-based deployment scenarios [67].
- These features supported use cases such as industrial automation, smart transportation, and electric power distribution, where deterministic delivery and fast recovery are important.

### 4.2. 3GPP Release 16

- Release 16 extends URLLC from baseline enablers toward phase-2 standardization in 5G, with a stronger emphasis on vertical-industry deployments, especially industrial automation [65].
- The main technical contributions include the following:
  1. Packet duplication and path redundancy mechanisms for reliability enhancement. In particular, 3GPP specified duplication across multiple paths using dual connectivity, two N3 and N9 tunnels, or two N3 tunnels, so that failure on one path would not interrupt packet delivery.
  2. Core-network quality-of-service enhancements such as packet delay budget (PDB) handling, dynamic PDB allocation, QoS monitoring, and session continuity improvements. These changes made latency guarantees more explicit at the service-management level.
  3. New radio-interface mechanisms including updated downlink control information formats, improved PDCCH monitoring, sub-slot HARQ-ACK feedback, dual HARQ-ACK codebooks, physical uplink channel refinements, and better multiplexing/prioritization of traffic with mixed latency requirements.
  4. Support for multiple active configured grants per bandwidth part, which improved uplink flexibility for periodic or bursty low-latency traffic.
- The associated study items were as follows: enhancement of URLLC support in the 5G Core network in SA2 [68], physical layer enhancements for NR URLLC in RAN [69], security of URLLC for 5GS [70], and EPC support for E-UTRAN URLLC in SA2 [67].

### 4.3. 3GPP Release 17

- Release 17 broadens URLLC evolution by integrating it more tightly with other 5G service requirements, including eMBB and mMTC, while continuing to improve low-latency behavior [65].
- The main technical contributions include the following:
  1. Further physical-layer improvements for HARQ-ACK and channel state information (CSI) reporting, which reduced feedback delay and improved uplink reliability [34].
  2. Enhanced intra-UE multiplexing and prioritization of traffic with different QoS priorities, allowing time-critical packets to be protected more effectively during scheduling [34].
  3. Improvements in edge computing support, including edge relocation capabilities, to reduce application-level response time when compute resources need to move closer to the user.
  4. URLLC operation in unlicensed spectrum, extending low-latency service support beyond licensed new radio (NR) deployments [34].
  5. Additional latency-oriented enhancements include NR sidelink, NR multiple-input multiple-output (MIMO) refinements, uplink data compression, and integrated access and backhaul duplexing improvements.

### 4.4. 3GPP Release 18

- Release 18 represents the start of the 5G-Advanced phase, where URLLC is increasingly shaped by automation, adaptation, and tighter service guarantees rather than only by radio-interface repetition mechanisms [65].
- The main technical contributions include the following:
  1. AI-assisted network optimization and energy-efficient operation, which improve scheduling responsiveness and resource utilization for latency-sensitive services [71].
  2. Integration with extended reality (XR) and multi-access edge computing, both of which require stable low-latency transport and benefit from tighter coordination between radio and compute resources [71].
  3. Introduction of Low Latency, Low Loss, and Scalable Throughput (L4S), which reduces queuing delay by allowing applications to adapt their sending rates more quickly and thereby improves latency under congestion [71].
  4. The *NR Timing Resiliency and URLLC* work item, which added support for timing synchronization and reporting in RAN, interworking with transport-layer time-sensitive networking (TSN), and feedback-based adaptation of downstream and upstream scheduling [72].

### 4.5. 3GPP Release 19 and Beyond

- Release 19 and later are less consistently documented under the explicit URLLC label, but they continue the evolution toward more robust, adaptive, and AI-assisted low-latency service support [71,73,74].
- The main technical directions include the following:
  1. Improved beam management and beamforming, including faster beam selection through UE-initiated measurement reporting, which can reduce interruption time in mobility and blockage-prone scenarios [71].
  2. Enhanced L1/L2-triggered mobility, with shorter service interruption times and improved dual-connectivity support across FR1 and FR2, which helps preserve service continuity for delay-sensitive devices [73,74].
  3. Continued use of AI/ML for RAN automation, network slicing optimization, timing synchronization, and split-RAN support, all of which indirectly strengthen URLLC performance by improving responsiveness and operational stability [71].

## 5. 5G URLLC Challenges and Enablers

This section discusses various causes and the approaches that have been used to improve the performance of URLLC. The majority of proposed URLLC approaches fall into two categories: either the method that reduces latency or the method that improves reliability. The types of wireless technology, mobility, network design, user/load density, and types of traffic are only a few of the many factors that affect latency and reliability [75,76]. We observe several different categories in which the sources of latency and reliability can be categorized, as stated in Table 5, described as follows:

### 5.1. Sources of Latency and Mitigation Approaches

Wireless communication involves multiple layers, each with distinct protocols. In the physical layer, the TTI is a fundamental unit of delay that accumulates across higher layers and significantly contributes to overall link latency. Key factors impacting latency in radio access networks include connection establishment (e.g., grant acquisition, random access), queuing delays, processing and computing delays, and retransmission delays. Beyond the RAN, other components that impact the overall E2E latency include the core network, data centers and cloud services, internet servers, and radio propagation delays. Various strategies have been developed to achieve low latency, focusing on factors such as channel access, radio resource management, radio slot design, and waveform design. The size of data blocks plays a critical role in determining network latency; achieving minimal delays requires the use of short codes, shorter TTIs, and faster HARQ retransmissions. Below, we outline various fundamental factors contributing to latency, along with methods to reduce and mitigate its effects:

- Frame Structure: The frame structure plays a fundamental role in achieving low latency in wireless communication systems. It determines how data is organized and transmitted over time, directly impacting the time-to-transmit and processing delays. To meet stringent latency targets, such as the 0.5 ms user-plane latency goal in 5G and beyond, flexible frame structures are employed that allow for shorter TTIs or mini-slots. These shorter time units reduce the amount of data processed and transmitted at once, thus lowering processing and scheduling delays. Additionally, adaptable parameters like subcarrier spacing, slot configuration, and uplink-downlink ratios enable dynamic adjustment of transmission times, allowing the system to respond quickly to traffic demands [77]. Fast turnaround of CSI and reduced or eliminated HARQ feedback time further contribute to minimizing latency by enabling quicker retransmissions and maintaining reliable communication [35]. Overall, a highly flexible and fine-grained frame structure is essential for enabling low-latency, high-reliability wireless services that coexist with other traffic types such as eMBB and mMTC.
- Propagation: Propagation latency, also known as RTT, is the time required for a signal to travel from a source to a destination and back. While fundamentally constrained by the laws of physics (speed of electromagnetic waves) and the distance between communicating parties, several strategies can be employed to mitigate its effects, such as minimizing the physical distance or optimizing the transmission environment. Edge computing, which involves deploying computing resources close to end users, reduces the amount of data that needs to travel long distances [78]. Caching frequently accessed data or content near users minimizes the need for repeated long-distance data fetching [79]. Peer-to-peer architectures can enable more direct communication between users, reducing reliance on central servers [80]. Effective propagation conditions, such as line-of-sight (LoS) communication and minimal multipath fading, contribute to lower retransmissions and higher reliability, which in turn reduce overall latency. Advanced techniques such as beamforming improve signal quality and enhance effective communication coverage by compensating for propagation impairments. In addition, millimeter-wave (mmWave) communications provide large bandwidths and high data rates, although they require directional transmission and dense deployments to mitigate their inherent propagation loss and blockage sensitivity. Furthermore, propagation-aware resource allocation and scheduling can adapt transmissions to real-time channel conditions, ensuring prompt delivery and minimizing delay. In dense or obstructed environments, deploying small cells or using non-terrestrial networks (NTNs) can mitigate long propagation delays.
- Processing: Processing delay refers to the time required to handle data or tasks. Major contributors include channel estimation, encoding, and decoding [81]. To reduce this delay, systems can employ rapid channel estimation for adaptive modulation and coding that optimizes data transmission for current channel conditions, ensuring that the transmitted data matches the current channel quality and reducing the need for retransmissions. Implementing advanced error correction and redundancy algorithms can reduce the need for re-transmitting lost or corrupted packets, thereby decreasing processing time due to retransmission delays [82]. Offloading processing tasks to nearby edge servers and breaking down tasks into smaller, parallelizable units can significantly reduce processing time [83]. Predictive algorithms can anticipate the next communication needs based on user behavior and initiate data transmission or processing proactively, reducing perceived latency.
- Retransmission: Reducing retransmissions is a key aspect of achieving low-latency URLLC. Retransmissions occur when a transmitted packet is lost or corrupted and needs to be retransmitted, causing additional delay in the network. HARQ in the access network combines automatic repeat requests (ARQs) with error correction coding, allowing the receiver to request retransmission of only missing or erroneous parts of a packet. This minimizes the amount of retransmitted data and reduces latency compared to a full retransmission. By implementing adaptive modulation and coding techniques that match the transmission rate to the channel quality, the likelihood of errors and retransmissions can be reduced [84]. Early HARQ feedback using predictive ARQ (PARQ) can anticipate potential packet loss and initiate preemptive retransmission, avoiding the delay associated with waiting for negative acknowledgments (NACK) [85]. On the other hand, shorter HARQ TTIs enable faster feedback on channel conditions, allowing quicker retransmission decisions. Using an adaptive modulation and coding scheme (MCS), which adapts the transmission rate to the channel quality, the potential errors and retransmissions can be reduced.
- Scheduling: Scheduling allocates shared channel access among users. Thus, multiple users or devices sharing a limited communication channel or resources induce latency from contention for resources, queuing delays, scheduling overhead, and interference management in communication systems with shared channels. Inefficient scheduling decisions can lead to unpredictable delays, collisions, interference, and increased retransmissions in dynamic environments. To reduce latency, several strategies are necessary, including optimized scheduling algorithms that prioritize low-latency traffic [86], dynamic adaptation of scheduling parameters to real-time network conditions, predictive techniques for proactive adjustments based on traffic patterns [87], and QoS-based traffic prioritization. These approaches ensure timely and efficient resource allocation while minimizing processing complexity and overhead.

### 5.2. Sources of Reliability Degradation and Mitigation Approaches

Reliability in radio links is generally measured in terms of BLER or the signal-to-interference-plus-noise ratio (SINR). Hence, a URLLC needs to experience SINR or BLER levels above or below a specified threshold, respectively, with a high degree of certainty. The primary factors affecting the reliability are collisions, coexistence, interference, Doppler shifts, synchronization, out-of-date channel state information, fading, dynamic channel conditions, or delayed packet delivery. At the physical layer, reliability aims to achieve low block error rates, which are dependent on elements such as error detection codes, channels, modulation approaches, diversity, retransmission procedures, etc. Reliability at the network level includes automated ARQ, and at the medium access layer, HARQ. Here, we list various fundamental factors that contribute to reducing reliability, along with methods to lower and alleviate its effects:

- Fading: The main factors in fading are multipath propagation, signal interference, and changing environmental conditions, which cause fluctuations in signal strength and quality [88]. Diversity techniques are like having a backup plan for your data: sending extra copies in different dimensions (space, frequency, or time) increases the chances of successful reception [89]. Sending the duplicated data via antenna diversity for fast fading, higher order single-user MIMO (SU-MIMO) diversity to lower the risk of encountering deep fades, inter-cell non-coherent joint transmission to recover from shadow fading, and cell failures are some of the examples of spatial diversity. In the same way, sending the data over multiple frequencies or time slots can help reduce the effect caused by fading. Techniques such as frequency hopping, orthogonal frequency division multiplexing (OFDM), and time division multiplexing (TDM) distribute the risk of fading and can improve resistance to fading-induced errors. In addition, channel predictive models can help anticipate fading variations and adjust data transmission accordingly, mitigating the effects of sudden signal degradation [90]. Relaying and beamforming are other approaches that reduce the impact of fading.
- Packet loss: Packet loss happens when data packets do not reach their destination. This can occur for many reasons. When the network is very busy, packets drop because the system reaches its capacity. Signals can also be interrupted by other devices or weakened by multipath fading, leading to errors [89]. Collisions occur when multiple devices try to send data simultaneously on a shared channel, resulting in more lost packets. Packet loss can also result from jitter, buffer overflows, QoS rules, packet corruption, routing problems, or network delays [91]. To reduce packet loss, networks require careful planning, reliable hardware, effective error-correction methods, and robust congestion control. Techniques such as multi-connectivity, time diversity, and frequency diversity, combined with HARQ, help improve data transmission stability.
- Interference: Interference happens when unwanted signals disrupt communication between the sender and receiver. This can be caused by nearby devices or external electromagnetic sources operating in the overlapping frequency band [91]. There are several types of interference, such as adjacent channel, co-channel, and cross-technology interference. Multipath propagation, where signals bounce, reflect, and scatter, is a major source of interference (constructive or destructive) at the receiver [92]. Transmitter proximity, signal reflection, frequency congestion, receiver sensitivity, etc., can also cause interference. To reduce interference, the network must use strategic frequency planning, dynamic spectrum management, power control mechanisms, and cognitive radio techniques to adapt according to the transmission parameters. Additionally, interference cancellation, spatial separation, directional antennas, and avoidance algorithms also help to enhance reliability for URLLC applications.
- Network configuration: If network configuration settings are inaccurate, this can result in poor performance, delays, and packet loss because resources and QoS parameters are not set correctly. Without proper monitoring and maintenance, problems may go unnoticed, leading to downtime and unreliable service. Poor load balancing can cause bottlenecks when traffic is high, which lowers reliability [93]. Weak security settings can allow unauthorized access, data breaches, and service interruptions. Not having redundancy or failover systems makes the network vulnerable and creates single points of failure. To improve wireless communication reliability, it is important to plan the network carefully, set parameters accurately, monitor regularly, maintain the system, and follow best practices for configuration [94].

While the techniques discussed above provide theoretical mechanisms to reduce latency and improve reliability, their practical deployment in real-world 5G/6G systems remains highly challenging. Approaches such as reduced TTI, preemption scheduling, multi-connectivity, and short HARQ RTT introduce significant scheduling overhead, signaling complexity, and processing burdens at the base station, particularly in heterogeneous environments that mix URLLC, eMBB, and mMTC traffic. Furthermore, their effectiveness is limited by imperfect CSI, synchronization errors, hardware constraints, and finite computational resources. Beyond the radio access network, mobility-induced channel variations, frequent handovers, dynamic interference conditions, fronthaul/backhaul delays, edge/cloud processing latency, and virtualization overhead all contribute to end-to-end performance degradation that is often overlooked in theoretical analyses. To bridge this gap, recent industrial testbeds, such as the 5G-ACIA [95] endorsed testbeds at Bosch and Ericsson, and the ETRI [96] industrial IoT testbed, have simulated high-density manufacturing floors to verify microsecond-level synchronization. Furthermore, the 5G Test Network Finland [97] supports research and validation for 5G and beyond technologies. Ultimately, these frameworks provide the foundational field trials necessary to de-risk wireless dependencies within smart manufacturing, autonomous transportation, and mission-critical robotics. Nevertheless, deployment experiences across these sectors, including remote healthcare, demonstrate that achieving stringent URLLC targets requires the joint optimization of communication, computing, and networking infrastructures [98]. These studies highlight a persistent gap between theoretical latency–reliability objectives and achievable, real-world performance under dense traffic and large-scale connectivity conditions. Consequently, practical implementation and rigorous experimental validation remain critical research directions for future 5G-Advanced and 6G systems.

### 5.3. Latency–Reliability Trade-Off

URLLC must achieve both low-latency and ultra-high reliability at the same time, which is difficult because these requirements often conflict. This trade-off is particularly significant because of how wireless channels operate and the constraints they impose on data transmission [2]. We discuss some conflicting factors that help improve one aspect, but at the cost of another.

Retransmission improves reliability by sending packets again when they are lost or damaged, ensuring data integrity. However, this process increases network RTT because extra time is needed to deliver the retransmitted packets. Control messages like ARQ and HARQ acknowledgments can slow things down even more. Thus, balancing reliability and latency is crucial, especially in time-sensitive applications. To better understand the relationship, consider a simple example where a user requests to send data at a rate of *R* with the application’s reliability criteria, $P_{rel}$. The transmission rate of the user *R* is the function of the transport block size (TBS) in accordance with the experienced SINR, and the number of retransmissions is $n_{max}$ that is needed to meet the application’s reliability requirements, $P_{rel}$. We can formulate *R* as follows:

(4) $$R=\frac{TBS(SNIR)}{T}{\left(1-BLER\right)}^{\left(n_{max}+1\right)}$$

where TBS is the number of data bits that can be transmitted in one RB, which depends on the MCS, and BLER is the target block error rate of the application. Reliability is the percentage of packets successfully received. To meet the required reliability $P_{rel}$ of the application, the number of retransmissions needed can be easily calculated by solving ${\left(1-BLER\right)}^{\left(n_{max}+1\right)}\geq P_{rel}$.

**Figure 3:**
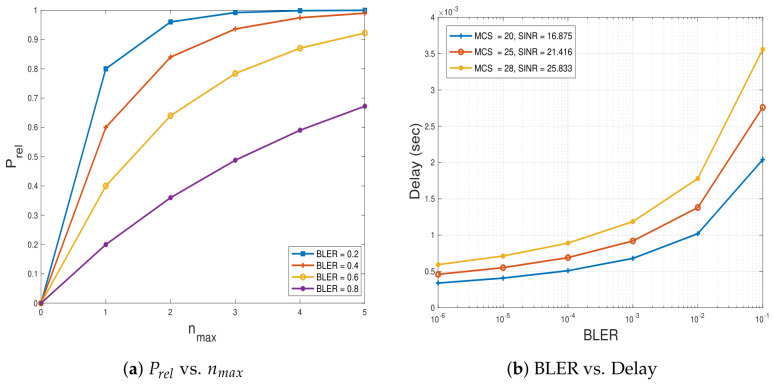
Reliability vs. latency trade-off.

The numerical analysis of Equation (4) reveals a clear trade-off between reliability and latency in wireless communication. Figure 3a shows the analytically derived improvement in reliability across various BLER levels as the number of retransmissions increases. However, Figure 3b demonstrates that achieving higher reliability through more retransmissions results in increased latency, especially for higher BLER values. This theoretical interplay highlights a fundamental challenge in wireless system design: maintaining reliability with higher BLER requires more retransmissions, leading to greater delays. The simultaneous degradation of BLER and latency stems from the retransmission-loop effect under fixed SINR. As shown in the equation, reducing *T* to maintain the target rate spikes the BLER. This spike forces the system to exhaust all retransmission attempts ($n_{max}$), causing latency to increase while reliability simultaneously drops because the success probability ${\left(1-BLER\right)}^{n_{max}+1}$ can no longer compensate for the poor channel conditions. This trade-off is crucial for URLLC applications demanding both ultra-high reliability and low latency, necessitating careful optimization of transmission parameters to balance these competing requirements.

Additionally, wireless channels are affected by interference, fading, and noise. Higher SNR improves reliability as it reduces the probability of errors in data transmission [99] but often requires sophisticated modulation schemes, potentially increasing latency (as we move from MCS 2O to MCS 28 as shown in Figure 3b). For instance, the spatial modulation scheme proposed in [100] requires full knowledge of CSI, which entails considerable pilot overhead and high-complexity channel estimation. While these schemes transmit more data per symbol, they introduce additional processing latency. Higher transmit power is another option for increasing signal SNR, but it can cause interference with nearby systems, leading to collisions and more retransmissions. Thus, balancing SNR, modulation complexity, and transmit power is crucial for optimizing both reliability and latency in wireless communications.

Furthermore, URLLC performance under various frame structures and scheduling mechanisms is evaluated. Two numerologies are considered with subcarrier spacing (SCS) values of 30 kHz and 60 kHz (μ = 1, 2), corresponding to slot durations of 0.5 ms and 0.25 ms, respectively. The system is evaluated over an SNR range of −5 to 15 dB with 2 dB steps, using 1000 packets per SNR point and a URLLC payload size of 256 bits per packet. QPSK modulation with AWGN channel modeling is employed in accordance with 3GPP NR low-latency assumptions. Two scheduling mechanisms are compared: dynamic scheduling with an average control-plane handshake delay of 1.75 slot durations, and grant-free (configured grant) transmission with zero scheduling delay. URLLC transmission utilizes mini-slot-based allocation with 4 OFDM symbols per transmission, and a processing delay of 0.15 ms is assumed at the transmitter and receiver. Reliability is evaluated in terms of BLER, while latency is computed as the sum of transmission, processing, scheduling, and retransmission delays under HARQ-based error recovery for both scheduling schemes.

**Figure 4:**
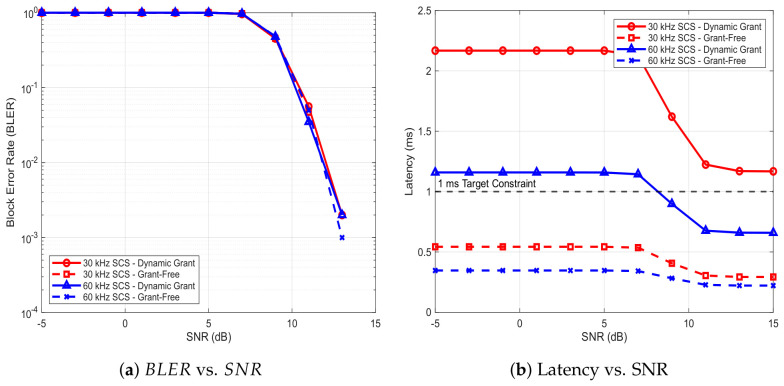
URLLC performance under various frame structures and scheduling mechanisms.

From Figure 4a,b, at low SNR values (−5 dB to 1 dB), the channel experiences a high BLER, resulting in frequent packet failures and triggering retransmission mechanisms. While retransmissions improve successful packet delivery, they introduce additional delay, thereby increasing the average end-to-end latency. This effect is particularly pronounced in the dynamic grant scheduling scheme, where each retransmission incurs extra scheduling and HARQ feedback delays. Conversely, the grant-free mechanism reduces latency by eliminating scheduling handshakes; however, reliability remains constrained by channel quality, especially under poor SNR conditions. As SNR increases, the BLER decreases rapidly, reducing the need for retransmissions and consequently lowering latency. In the high-SNR region (less than 8 dB), both latency and reliability improve simultaneously, approaching the ideal URLLC operating point characterized by sub-millisecond latency and near-zero BLER. The impact of numerology is also evident in the trade-off behavior: the 60 kHz SCS configuration provides shorter transmission opportunities and faster recovery from packet errors, enabling lower latency without sacrificing reliability. In contrast, the 30 kHz SCS configuration achieves similar reliability levels but at the cost of longer transmission durations and increased overall delay. Therefore, the trade-off is not solely determined by scheduling strategy but also by frame structure parameters such as subcarrier spacing and slot duration. The results indicate that grant-free scheduling combined with higher numerology shifts the latency–reliability operating curve toward a more favorable region, allowing stringent URLLC requirements to be satisfied with minimal delay while maintaining high transmission success probability.

Similarly, larger packet sizes can enhance data transmission efficiency and reduce overhead, but they are more susceptible to errors, such as burst errors, which occur when consecutive bits are corrupted due to noise or interference [101]. The retransmission of a large packet can significantly increase latency [102]. Conversely, fragmenting packets into smaller sizes may improve reliability but might add overhead and potentially increase latency. A wider channel bandwidth can increase the data rate and, consequently, reduce latency. However, a wider bandwidth might also make the wireless link more susceptible to interference and decrease reliability. In contrast, a narrower bandwidth offers better reliability, but at the expense of higher overhead and queuing latency [103].

In practical 5G/6G deployments, the end-to-end latency experienced by URLLC services is influenced not only by transmission delay but also by several implementation-level delay components that are often simplified in analytical models. One important contributor is scheduler delay, which arises from resource allocation decisions at the base station and becomes more significant under heterogeneous traffic coexistence. Similarly, HARQ feedback timing and retransmission delay can substantially affect latency, particularly when multiple retransmission attempts are required to satisfy strict reliability constraints. Beyond the radio access network, fronthaul and backhaul transport delays between distributed radio units, centralized units, and core network entities introduce additional latency variability, especially in cloud-based and virtualized architectures. Furthermore, edge/cloud processing delay, including packet inspection, AI-driven orchestration, and application-layer computation, may become a bottleneck for delay-sensitive services such as industrial automation and remote healthcare. These factors demonstrate that achieving ultra-low latency in practical deployments requires holistic optimization across both communication and computing infrastructures.

### 5.4. URLLC Coexistence

In modern 5G networks, the coexistence of URLLC with eMBB or eMTC or both is not only common but also essential to achieving the vision of a unified, flexible communication platform capable of serving a wide range of applications simultaneously. One such scenario can be found in a factory where a 5G base station is serving high-definition video (eMBB), real-time robotic control (URLLC), and IoT temperature sensors (eMTC) simultaneously. Each of these service categories has unique performance requirements and traffic characteristics. eMBB targets high data rates and large bandwidth, while eMTC (or mMTC) enables massive connectivity for low-power, low-data-rate IoT devices. However, having these services in one place creates serious technical challenges. The biggest issue is resource contention, as URLLC traffic needs immediate transmission and may interrupt ongoing eMBB data transfers, reducing broadband performance and potentially causing service disruptions. The QoS conflict between the services, where eMBB prefers high-capacity, delay-tolerant channels and URLLC needs short, time-sensitive bursts, makes scheduling and resource allocation extremely complex. On top of that, managing interference becomes harder in dense networks, especially since eMTC devices, which use low-power narrowband communication, are more susceptible to interference from high-power URLLC signals.

To address this, 5G systems employ flexible numerology and mini-slot transmission to shorten transmission time for URLLC [104], as well as preemption and puncturing mechanisms that allow URLLC packets to interrupt ongoing eMBB transmissions with minimal delay. On the uplink, grant-free and configured-grant mechanisms are introduced to eliminate scheduling latency, benefiting both URLLC and mMTC devices [105]. At the PHY and MAC layers, technologies such as massive MIMO [106], non-orthogonal multiple access (NOMA) [107], and rate-splitting multiple access (RSMA) [108] enable multiple services to share spectrum efficiently through spatial, power, or code-domain multiplexing.

Mathematically, this resource contention can be framed as a joint utility maximization problem over a shared system bandwidth *B*. When sporadic URLLC traffic punctures an ongoing eMBB transmission within a macro-slot of duration $T_{e}$, the resulting effective signal-to-interference-plus-noise ratio (SINR) of the eMBB user ($\gamma_{e}$) drops significantly and is modeled as:

(5) $$\gamma_{e}=\frac{P_{e}{\left|h_{e}\right|}^{2}}{\left(1-\alpha \right)I_{\mathrm{inter}}+\alpha P_{u}{\left|h_{u}\right|}^{2}+\sigma^{2}}$$

where $P_{e}$ and $P_{u}$ denote the transmit powers of the eMBB and URLLC services, $h_{e}$ and $h_{u}$ represent their respective channel gains, $\sigma^{2}$ is the thermal noise variance, and α∈{0,1} indicates the binary puncturing state. Under a Poisson arrival rate of URLLC packets ($\lambda_{u}$) and a mini-slot duration $T_{u}$, the total throughput loss ($\Delta R_{e}$) experienced by the coexisting eMBB service is quantitatively derived as $\Delta R_{e}=B\log_{2}\left(1+\gamma_{e}\right)\times \left(1-\exp\left(-\lambda_{u}T_{u}/T_{e}\right)\right)$. Conversely, for mMTC devices, the coexistence challenge is bounded by finite blocklength theory (N≤100 symbols) where the achievable rate $R_{\mathrm{mmtc}}$ is strictly constrained by the channel dispersion *V*:

(6) $$R_{\mathrm{mmtc}}\approx \log_{2}\left(1+\gamma_{m}\right)-\sqrt{\frac{V}{N}}Q^{-1}\left(\epsilon \right)$$

where $\gamma_{m}$ is the mMTC SINR, ϵ represents the strict target block error rate ($\mathrm{BLER}=10^{-5}$), and $Q^{-1}\left(\cdot \right)$ is the inverse Gaussian Q-function. This mathematical formulation quantifies the severe reliability and capacity penalties incurred across the entire resource grid when time-sensitive, high-power URLLC bursts force the network away from the classical Shannon capacity boundary.

**Figure 5:**
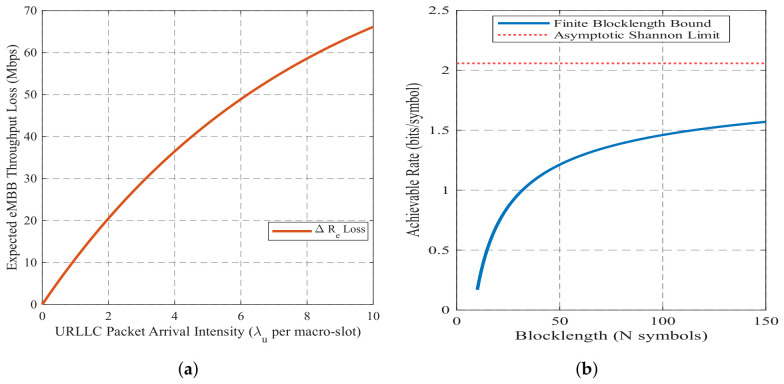
Coexistence analysis. (**a**) eMBB Throughput Loss vs. Arrival Intensity; (**b**) mMTC Finite Blocklength Capacity Bound.

Quantitative analysis of heterogeneous service coexistence under URLLC resource contention is illustrated in Figure 5a,b with simulation parameters from Table 6. Figure 5a shows the expected throughput penalty ($\Delta R_{e}$) experienced by eMBB users due to preemptive URLLC puncturing. As the URLLC arrival intensity $\lambda_{u}$ increases, the probability of puncturing ongoing eMBB transmissions also increases, resulting in higher throughput degradation caused by resource preemption and interference. This highlights the trade-off between stringent URLLC latency requirements and eMBB spectral efficiency.

Figure 5b presents the finite-blocklength performance for mMTC communication. For small blocklengths *N*, the achievable rate remains significantly below the asymptotic Shannon limit due to the additional coding redundancy required to satisfy strict reliability constraints. As the blocklength increases, the achievable rate gradually approaches the Shannon capacity. These results demonstrate the inherent trade-off among latency, reliability, and transmission efficiency in short-packet communications. Overall, the analysis confirms that efficient coexistence of URLLC, eMBB, and mMTC services requires careful cross-layer resource management to balance latency, reliability, throughput, and scalability in future 5G-Advanced and 6G networks.

Moreover, at the network level, dynamic network slicing and AI-driven resource orchestration allow the network to allocate bandwidth, computing, and latency budgets adaptively to different service slices [109]. Machine learning and deep reinforcement learning (DRL) are increasingly used for predictive scheduling, allowing the network to anticipate URLLC arrivals and optimize resource allocation without compromising eMBB throughput or mMTC scalability. Table 7 summarizes the various approaches for the coexistence of URLLC with eMBB and/or mMTC. Despite these advances, significant trade-offs remain between reliability, latency, spectral efficiency, and complexity.

## 6. The Vision of URLLC Beyond 5G

Despite growing interest in URLLC, its core principles remain poorly understood, especially as new high-stakes applications demand greater reliability, latency, connectivity, and scalability. These challenges are driving the evolution of mission-critical applications beyond 5G into 6G. The vision for next-generation URLLC includes achieving extreme reliability and ultra-low latency, potentially reaching microsecond-level latency, and supporting massive connectivity by integrating URLLC with mMTC for smart cities and industrial automation. Moreover, 6G will introduce broadband URLLC, using AI and edge computing for real-time optimization. These advances will enable emerging technologies such as haptic communications and support critical applications such as autonomous systems and XR. At the same time, 6G will ensure URLLC coverage is global and seamless [21,22].

**Figure 6:**
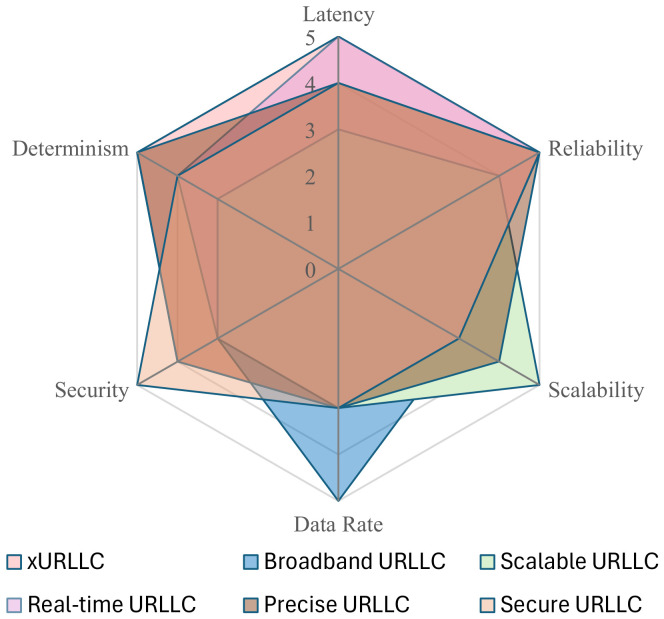
URLLC service categories in 6G.

Figure 6 presents a comparative analysis of emerging 6G URLLC service modes across key performance indicators, highlighting the trade-offs among latency, reliability, scalability, data rate, security, and determinism. Similarly, Table 8 summarizes the emerging URLLC service categories and their associated design objectives. Although all future URLLC modes share the common goal of ultra-low-latency and ultra-high reliability, they differ in terms of bandwidth demand, scalability, determinism, intelligence, precision, and security requirements. To improve clarity, the discussion is organized into six categories: extreme URLLC, broadband URLLC, scalable URLLC, real-time URLLC, precise URLLC, and secure URLLC. Each category targets distinct application domains and introduces unique technical challenges and enabling technologies.

### 6.1. Extreme URLLC

Extreme URLLC (xURLLC) extends the objectives of 5G URLLC by pushing reliability, latency, and scalability to unprecedented levels in order to support mission-critical applications such as advanced industrial automation, autonomous transportation systems, and remote healthcare. These applications require sub-millisecond latency and reliability levels beyond 1 − 10^−5^ [21].

Achieving such stringent requirements significantly increases the complexity of both radio access and core network operations. Delay must be minimized across all layers, including the physical, MAC, network, and backhaul layers. Extremely short transmission intervals make long coding schemes and multiple retransmissions impractical, thereby complicating error control mechanisms. Furthermore, wireless impairments such as fading and interference must be handled within microsecond-level timescales, while network nodes are required to process and deliver packets almost instantaneously [121]. Resource sharing with eMBB and mMTC services further complicates reliability guarantees under heavy traffic conditions.

To address these challenges, 6G networks rely on advanced coding techniques, network slicing, edge intelligence, and proactive resource allocation. Machine learning enables real-time prediction, anomaly detection, and adaptive optimization across communication layers [122]. Robustness can be further improved through multimodal sensing approaches that combine radio communication with vision and LIDAR systems to mitigate environmental uncertainty and sudden failures [123,124]. In parallel, communication and control co-design is emerging as a key paradigm for supporting massive numbers of connected devices without compromising extreme performance guarantees [21].

### 6.2. Broadband URLLC

Broadband URLLC integrates the ultra-reliable and low-latency characteristics of URLLC with the high-data-rate capabilities of eMBB to support bandwidth-intensive and delay-sensitive applications. Representative use cases include 4K/8K video streaming, holographic communication, immersive six degrees of freedom (6DoF) experiences, and haptic-enabled remote surgery [125]. These applications require not only high transmission speeds but also continuous and synchronized data delivery.

To support large packet transmissions without excessive decoding complexity, broadband URLLC depends on improved spectral efficiency, wider bandwidth availability, and adaptive modulation and coding schemes [22,25]. The use of mmWave and terahertz frequency bands enables significantly higher data throughput by providing broader transmission bandwidths [22]. However, operation at these high frequencies introduces severe path loss, fading, coverage limitations, and increased interference management complexity.

To maintain both high throughput and strict reliability, broadband URLLC employs intelligent scheduling, advanced multiple-access techniques, edge computing, and streamlined processing pipelines. Adaptive resource allocation dynamically balances network resources among heterogeneous traffic flows, while interference management techniques help preserve communication stability and quality of service under heavy network load [126].

### 6.3. Scalable URLLC

Scalable URLLC addresses the growing demand for ultra-reliable and low-latency connectivity among massive numbers of IoT devices in applications such as smart manufacturing, industrial automation, and healthcare monitoring. Compared with 5G mMTC, scalable URLLC significantly increases connection density while maintaining millisecond-level latency and high reliability [127].

The primary challenge in scalable deployments arises from network complexity, resource contention, heterogeneous traffic requirements, and dynamic topology changes. Efficient management of CSI overhead and the deployment of lightweight, energy-efficient machine learning models are particularly important for resource-constrained IoT devices [22,128].

To overcome these challenges, scalable URLLC leverages edge and fog computing architectures to process data closer to end devices, thereby reducing latency and alleviating backhaul congestion [128]. Software-defined networking (SDN) and network function virtualization (NFV) further improve flexibility, resource orchestration, and delay management. In addition, intelligent scheduling, wideband resource allocation, and multi-connectivity techniques help sustain scalability, reliability, and communication efficiency across dense IoT deployments.

### 6.4. Real-Time URLLC

Real-time URLLC in 6G extends beyond conventional packet delivery by emphasizing the freshness and relevance of information required for interactive physical, digital, and biological systems [129,130]. Unlike 5G URLLC, which primarily focuses on transmission delay, 6G real-time URLLC prioritizes timely and actionable information exchange for applications such as mixed-reality telepresence, autonomous driving, and in-body medical monitoring.

This paradigm introduces the Age of Information (AoI) metric, which measures the time elapsed since the most recent data update and provides a more meaningful indicator of system responsiveness and situational awareness [118]. To support these applications, 6G targets latency below 0.1 ms together with near-perfect reliability reaching 99.999999%. Achieving these goals requires technologies such as edge intelligence, network slicing, semantic communication, and AI-driven resource optimization.

A major challenge in real-time URLLC is finite blocklength communication, since extremely short packets must simultaneously satisfy stringent latency and reliability constraints. Consequently, new coding, modulation, and scheduling strategies are required to optimize AoI while balancing reliability, throughput, and energy efficiency. Cross-layer co-design across the physical, MAC, and network layers is also essential to maintain information freshness and robustness in highly dynamic environments. Furthermore, future network models must incorporate not only timing constraints but also the semantic importance of information for decision-making processes.

### 6.5. Precise URLLC

Precise URLLC enhances traditional URLLC by delivering highly accurate, synchronized, and deterministic information streams suitable for critical control applications [31,131,132]. This category targets applications such as industrial automation, remote telesurgery, and autonomous vehicle platooning, where timing jitter, packet loss, or throughput fluctuations may compromise safety and operational stability.

To address these requirements, precise URLLC introduces strict performance guarantees, including lossless networking, deterministic throughput, bounded latency, in-time delivery, and on-time synchronization with control cycles [133]. These guarantees ensure that communication remains predictable, synchronized, and tightly aligned with system dynamics.

Realizing precise URLLC requires deterministic communication mechanisms such as reserved resources, strict scheduling, and time-sensitive networking. Cross-layer optimization across the physical, MAC, network, and application layers is essential to minimize latency variation, jitter, and throughput instability. In addition, novel coding schemes, diversity techniques, redundancy mechanisms, and application-aware resource orchestration are necessary to maintain deterministic performance in mission-critical control loops.

### 6.6. Secure URLLC

Secure URLLC, also referred to as hyper-reliable low-latency communication (HRLLC), introduces stringent security requirements while maintaining ultra-low-latency and extremely high reliability [120,134]. Traditional cryptographic methods often impose excessive computational overhead and latency, making them unsuitable for mission-critical real-time applications [120].

To address these limitations, 6G secure URLLC incorporates lightweight security mechanisms directly into the communication architecture. Physical layer security (PLS) exploits the randomness and dynamic nature of wireless channels to enable secure communication and key generation with minimal computational complexity [25]. AI-driven threat detection and proactive cybersecurity mechanisms further enhance network resilience and adaptability.

Moreover, 6G security architectures are expected to adopt zero-trust principles to continuously verify devices, users, and interactions across the Internet of Everything (IoE). To prepare for future quantum computing threats, secure URLLC also integrates post-quantum cryptography (PQC) techniques capable of resisting quantum-enabled attacks.

## 7. Future Direction

Achieving true URLLC in B5G systems and pushing into the 6G era presents a rich landscape for future research, moving beyond isolated performance metrics toward holistic system integration. This evolution mandates a transition from merely connecting devices to creating an “Intelligent Network of Everything” that is inherently resilient, adaptive, and trustworthy.

**Integration of AI and Machine Learning:** The future of URLLC beyond 5G and toward 6G networks will be shaped significantly by the integration of AI/ML technologies. These advances will enable networks to dynamically optimize resources, forecast traffic congestion, and detect security threats in real time. As a result, these networks can adapt intelligently to heterogeneous and dense communication environments. Particularly, the federated learning (FL) technique uses decentralized decision-making at the edge while preserving the privacy of the data collected at the edge device. Moreover, self-healing network capabilities, which are driven by AI/ML, will enhance reliability while at the same time minimizing downtime in mission-critical systems.
**Advancements in Physical Layer Technologies:** Parallel to AI integration, breakthroughs in physical layer technologies are essential to meet stringent latency and reliability demands. THz and sub-THz communications will unlock exceedingly wide bandwidths critical for emerging high-data-rate applications like holographic communication and massive digital twins [28,29,41]. Complementing this, ultra-massive MIMO techniques and RIS will provide improved spectral efficiency and the ability to actively manipulate wireless propagation environments. These advances, together with novel coding and modulation schemes designed for short-packet URLLC traffic, will help overcome the fundamental latency–reliability trade-off.
**Security and Privacy Enhancements:** Security and privacy will continue to be top priorities in future URLLC systems. Using a lightweight cryptographic technique that introduces minimum latency overhead will be crucial, along with new physical-layer security techniques that use channel characteristics to safeguard data confidentiality and integrity. Moreover, the introduction of quantum computing demands the use of quantum-resistant cryptography. Furthermore, zero-trust architectures can keep networks secure by constantly verifying devices and users, which is especially important as the IoE continues to grow and introduce new vulnerabilities.
**Edge Computing and Network Slicing:** Edge computing and network slicing will continue to grow, bringing processing and storage closer to the end users. This helps minimize end-to-end latency and makes it easier to deliver the specific quality of service each application needs. The adoption of blockchain and other distributed ledger technologies will enhance decentralized trust and accountability that are critical for multi-provider and heterogeneous network environments. These technologies will enable fine-grained isolation of URLLC services and support complex application requirements.
**Standardization and Testing:** To make these technologies work efficiently, the standardization ecosystem needs to grow and adapt. It must support interoperable protocols that allow different systems to work together smoothly. Strong testing methods like digital twins will be needed to evaluate performance in realistic conditions, along with unified KPIs to clearly measure the broader goals of URLLC. Cross-industry collaboration will be vital to align network capabilities with application-specific needs and accelerate deployment.
**Ubiquitous Coverage through Non-Terrestrial Networks (NTNs):** Non-terrestrial networks (NTNs) play a vital role in improving URLLC coverage. They bring together LEO satellites, high-altitude platforms (HAPs), and UAVs with regular ground networks. This seamless integration solves connectivity problems, especially in remote locations where there is weak coverage. Some of the main challenges, such as propagation delay, synchronization, and multi-domain coordination, must be managed to meet the stringent latency and reliability demands of URLLC.
**Convergence of Communication, Computing, and Control (3C):** Finally, URLLC will no longer be isolated to communication alone; it will converge with computing and control to form integrated frameworks essential for real-time cyber-physical applications such as cooperative robotics, remote surgery, and smart energy grids. This convergence requires harmonious sensing, communication, computation, and actuation under tight timing and reliability constraints.

Addressing these complex and interdisciplinary challenges in a clear and coordinated manner is essential. This will unlock the full potential of URLLC in future intelligent and hyper-connected network environments. These networks will form the foundation for many emerging societal and industrial applications.

## 8. Conclusions

URLLC is a key 5G technology that enables mission-critical applications such as factory automation and remote surgery. As we move toward 6G, expectations for reliability, latency, scalability, and coexistence with other 5G services grow even tougher. These demands require current system architectures to evolve and call for new solutions. This work presents a comprehensive overview of 5G URLLC, identifying existing limitations and the technological advancements designed to address them. Considering emerging innovations, ongoing standardization efforts, and mission-critical use cases, it shows that latency, reliability, and coexistence are closely connected and that improving one often affects the others. The insights presented here point toward what URLLC may look like in 6G and beyond, emphasizing the growing importance of AI-driven orchestration, smarter resource management, and advanced PHY/MAC techniques to meet the extreme requirements of future ultra-reliable, low-latency applications.

## Figures and Tables

**Table 1:** Examples of 6G URLLC use cases beyond 5G capabilities [1,2,22,33].

Use Case	Description	Beyond 5G Capabilities
Remote Telemedicine & Surgery	Real-time robotic surgery with haptic feedback and imperceptible latency	Microsecond latency, enhanced reliability, precise synchronization
Autonomous Vehicles	Collaborative driving with thousands of sensors per vehicle, ultra-reliable control	Sub-millisecond latency, massive device density, AI-enabled mobility
Industrial Robotics	Ultra-precise robotic coordination with synchronized operations	Microsecond-level timing precision, zero error tolerance
Mixed Reality & Holography	Immersive interaction with real-time rendering and minimal latency	Terabit-per-second data rates, ultra-low latency for seamless experience
Defense & Emergency Communications	Mission-critical connectivity in extreme scenarios	Near-perfect reliability and latency under harsh conditions
Massive Digital Twins & Smart Cities	Real-time monitoring and control of complex infrastructures	Massive data throughput and synchronization
AI-Driven Network & Edge Computing	Real-time AI offloading and adaptive network optimization	Integration of AI/ML for dynamic resource allocation and prediction

**Table 2:** Comparison of existing URLLC surveys with the proposed work.

Survey	Coverage	Analysis	eMBB/mMTC Coexistence	3GPP Standardization	Practical Deployment	Main Focus/Limitation
Sutton et al. [1]	5G	PHY/MAC Focus	Limited	Partial	No	Focuses mainly on enabling PHY/MAC techniques for URLLC.
Ji et al. [35]	5G	PHY Layer	No	Limited	No	Discusses ultra-reliable transmission techniques with limited system-level analysis.
Lee et al. [34]	5G	PHY/MAC	Limited	Partial	No	Emphasizes physical-layer enhancements and radio resource techniques.
Liu et al. [39]	5G	Application-Specific	No	No	Limited	Focuses mainly on vertical applications and use cases.
Haque et al. [25]	5G/6G	Partial	Qualitative	Partial	Limited	Provides a broad overview but limited quantitative coexistence and deployment analysis.
Shaik et al. [37]	5G/6G	AI-Centric	No	No	Limited	Focuses on AI/ML-based optimization techniques for URLLC.
Pradhan et al. [38]	5G/6G	Security focus	No	No	Limited	Primarily addresses security and privacy aspects of URLLC.

**Table 3:** Latency and reliability requirements for URLLC devices and applications [55].

Device/Use Case	References	Latency	Reliability	Comments/Remarks
Industrial robot	[24]	<1 ms	>99.999%	Precise and safe real-time control in robotics and automation lines.
Mobile robot	[24]	<1 ms	>99.9999%	Flexible manufacturing, immediate adaptation, and safety in mobile logistics.
Remote healthcare and surgery	[26]	<100 ms	>99.9999999%	Responsive remote diagnostics or assistance in medical interventions.
Sensors	[56]	∼100 ms	>99.99%	Periodic, non-critical monitoring for environmental or equipment parameters.
Head-mounted display	[57]	<10 ms	>99.999%	Supports AR/VR in industrial and medical settings for immersive user experience.
Automated guided vehicles	[58]	<10 ms	>99.9999%	Safe, remote, and precise material transport in smart factories.
Security camera	[53]	∼100 ms	>99.99%	Infrastructure surveillance and event documentation, tolerates higher latency.
V2X (Platooning)	[53]	<3 ms	>99.999%	Strict real-time vehicular coordination for collaborative driving.
V2X (Cooperative maneuver)	[53]	<10 ms	>99.999%	Vehicle-to-vehicle communication for traffic safety and flow optimization.
Remote surgery	[53]	<10 to 100 ms	>99.9999%	Critical tactile Internet applications (e.g., telemedicine, remote robotics).

**Table 4:** Evolution of URLLC-related standardization from Release 15 to Release 19.

Release	Main Focus	Representative Technical Contributions
15	Initial URLLC enablers	Short TTI, grant-free transmission, HARQ timing reduction, PDCCH repetition, PDCP duplication, improved access recovery, EPC support for E-UTRAN URLLC
16	Phase-2 URLLC for verticals	Packet duplication across multiple paths, QoS/PDB control, improved PDCCH monitoring, sub-slot HARQ-ACK, dual codebooks, configured grant enhancements
17	Broader 5G integration	HARQ-ACK/CSI feedback improvements, intra-UE prioritization, edge relocation, URLLC in unlicensed spectrum, sidelink and uplink compression enhancements
18	5G-Advanced URLLC	AI-assisted optimization, XR and MEC integration, L4S, timing resiliency, TSN interworking, feedback-based scheduling adaptation
19+	Adaptive and mobility-aware support	Beam management improvement, enhanced mobility, shorter interruption time, AI/ML-based RAN automation and slicing optimization

**Table 5:** Source and approaches to reduce latency and increase reliability.

Objective	Component	Description	Approaches
Latency	Frame structure	Time required to send each packet of request, grant, or data.	TTI size ensures quick payload transfer. Reduced slot and mini-slot transmission. Grant free access
	Propagation	The duration for a signal to move from the transmitter to the receiver.	D2D and relay achieve lower queuing delay Cellular can achieve higher throughput. Zero latency approach
	Processing	Channel estimation, Signal processing (e.g., encoding and decoding).	Appropriate modulation and coding. Offloading task Advanced error correction
	Retransmission	Packet retransmission in access network. HARQ process delay for each retransmission.	Short HARQ round-trip time (RTT) to allow retransmissions within the latency budget. Early HARQ feedback and automatic HARQ retransmissions.
	Scheduling	Minimize queuing delays due to congestion, propagation, and retransmission.	Low-latency payloads were given priority scheduling to reduce BS queue delays. Preemption scheduling for high-priority data over low-priority traffic without radio resource reservation
Reliability	Fading	Attenuation of a signal due to time, geographical position, and radio frequency.	Antenna diversity data duplication for fast fading. Deep fades are less likely with higher-order SU-MIMO diversity. Joint transmission between non-coherent cells recovers from shadow fading and cell failures.
	Packet loss	Transmitted data does not reach its intended destination due to congestion, device failure, firewalls, etc.	Retransmission with time diversity and frequency diversity using HARQ. Multi-connectivity.
	Interference	Noise in a signal, such as co-channel interference, adjacent channel interference	Receivers for minimizing interference (e.g., MMSE-IRC or NAICS). Interference coordination between cells.
	Network configuration	Assigning network settings, policies, flows, and controls.	Service-specific link adaptation. Preemptive scheduling.

**Table 6:** Simulation parameters for URLLC–eMBB–mMTC coexistence analysis.

Parameter	Value
System bandwidth (*B*)	20 MHz
eMBB macro-slot duration ($T_{e}$)	1 ms
URLLC mini-slot duration ($T_{u}$)	0.125 ms
Baseline eMBB SNR (${\mathrm{SNR}}_{e}$)	20 dB
Baseline URLLC SNR (${\mathrm{SNR}}_{u}$)	25 dB
mMTC SNR (${\mathrm{SNR}}_{m}$)	5 dB
URLLC arrival intensity ($\lambda_{u}$)	0–10 packets/slot
Finite blocklength (*N*)	10–150 symbols
Target BLER (ϵ)	$10^{-5}$
eMBB throughput model	Shannon-based
mMTC rate model	Finite blocklength approximation
Puncturing mechanism	Preemptive URLLC scheduling
Channel assumption	AWGN channel

**Table 7:** Problems and solution approaches for coexistence of URLLC with eMBB and/or eMTC.

Paper	Coexistence	Challenge	Approach
[110]	eMBB	Modeling coexistence trade-offs between URLLC and eMBB in resource allocation; inefficiency vs. latency/reliability conflicts	Comparative framework evaluating multiple resource sharing and priority strategies including preemption; shows preemption reduces resource needs
[111]	eMBB and mMTC	Heterogeneous 5G service integration; interference and resource isolation	Network slicing for service isolation; hybrid multiplexing and scheduling for QoS differentiation
[112]	eMBB	Inefficient resource utilization and delay in URLLC–eMBB coexistence	Joint channel selection and power allocation using NOMA, priority multiplexing
[113]	eMBB	Scheduling conflicts and resource contention in URLLC–eMBB coexistence	Puncturing-based co-scheduling with URLLC preemption to minimize eMBB degradation
[30]	eMBB	Dynamic adaptation to mixed traffic demands; balancing latency and throughput	AI/DRL-based predictive resource scheduling optimizing slice allocation
[114]	mMTC	Grant-free mMTC (massive access) can coexist with URLLC without harming URLLC latency/reliability	Grant-free coded random access + massive-MIMO separation + power/preamble design to enable non-orthogonal coexistence and reliable URLLC demarcation.
[115]	mMTC	Providing immediate, reliable, and low-latency access for URLLC traffic in massive IoT networks	Priority-based access scheme that enables preemptive and flexible resource allocation for URLLC traffic.

**Table 8:** Summary of new service mode of URLLC.

Categories	Features	Challenges	Approach	Paper
Xtreme URLLC	Faster and reliable data-driven predictions; Model and address rare events without sacrificing spectral efficiency; Relax the very stringent latency and reliability requirements through communication and control co-design	Predicting wireless environments (channels, interference, services, etc.) reliably based on past data samples; Transfer and fuse non-RF and RF modalities with minimum overhead; To relax URLLC requirements by taking into account control dynamics, while ensuring control stability	Machine learning, AoI, network slicing, mapping, localization	[21,22]
Scalable URLLC	Supports much higher connection density, bandwidth, data rate, and low-latency experience for IoT devices Tangible experiences with ultra-reliability and ultra-responsiveness	Design CSI acquisition solution exploiting statistical channel information in distributed systems serving a massive number of users Novel random access solutions for massive access with fast preamble resolution	Traffic prediction, joint user detection and decoding, localization, fingerprinting mapping, predictive RRM	[22,113]
Broadband URLLC	A realistic, volumetric and immersive sense of experience Communication with efficient MCS that optimizes spectrum usage. solutions Supports high data-rate using available bandwidth at millimeter wave and THz bands	Combating the fading selectivity of the channels for high modulation and coding order Reducing decoding complexity	Polar codes, graph and cluster-based decoders, genetic algorithms, reinforcement learning, non-binary codes	[22,116,117]
Real-time URLLC	Supports immediate communication, sensing, and control	To maintain up-to-date information under various conditions	AoI, Finite blocklength, Synergies between TSC and URLLC	[32,118]
Precise URLLC	Guarantees in network performance, including lossless networking, throughput guarantee, latency guarantee, in-time guarantee, and on-time guarantee	Ensuring that data transmission and reception occur with minimal delay and minimal error rates, meeting stringent requirements for precision and reliability	Maximum allowable transfer interval (MATI), the maximally allowable delay (MAD), AoI	[2,22,119]
Secure URLLC	Secure handling of small data packets Integration of AI/ML for network management Integrated sensing and communication (ISAC) [27]	Core latency vs. security trade-off (traditional crypto is too slow), an expanded attack surface across billions of diverse IoT devices, vulnerabilities in AI/ML systems to adversarial attacks, and the future threat posed by quantum computing to current encryption standards.	PLS for lightweight encryption, a ZTA for strict verification, adoption of PQC standards, and AI/ML for proactive threat detection	[25,120]

## Data Availability

Not applicable.
